# Supplementary material for: Microwave-induced conductance replicas in hybrid Josephson junctions without Floquet—Andreev states
Source: Nat Commun. 2023 Oct 26;14:6798. doi: 10.1038/s41467-023-42357-5 (PMC10603169; doi:10.1038/s41467-023-42357-5)
Supplement: Supplementary file 1 — Supplementary Information [file 41467_2023_42357_MOESM1_ESM.pdf]

Supplementary Information: Microwave-induced conductance replicas in hybrid Josephson junctions without Floquet–Andreev states

Daniel Z. Haxell,[1] Marco Coraiola,[1] Deividas Sabonis,[1] Manuel Hinderling,[1] Sofieke C. ten Kate,[1] Erik Cheah,[2] Filip Krizek,[1,2] Rüdiger Schott,[2] Werner Wegscheider,[2] Wolfgang Belzig,[3] Juan Carlos Cuevas,[3,4] and Fabrizio Nichele[1,*]

[1]*IBM Research Europe—Zurich, 8803 Rüschlikon, Switzerland*

[2]*Laboratory for Solid State Physics, ETH Zürich, 8093 Zürich, Switzerland*

[3]*Fachbereich Physik, Universität Konstanz, D-78457 Konstanz, Germany*

[4]*Departamento de Física Teórica de la Materia Condensada and Condensed Matter Physics Center (IFIMAC), Universidad Autónoma de Madrid, E-28049 Madrid, Spain*

CONTENTS

Supplementary Note 1: Frequency Dependence of Conductance Response 2

Supplementary Note 2: Spectroscopy at an In-Plane Magnetic Field 2

Supplementary Note 3: Microwave Field Strength from Shapiro Steps 3

Supplementary Note 4: Modelling of Photon Assisted Tunnelling Data 4

Supplementary Note 5: Removal of Background Conductance 5

Supplementary Note 6: Sum Rule for Conductance Replicas 7

Supplementary Note 7: Microwave Coupling Strength from High-Bias Conductance 7

Supplementary Note 8: Results for $V_{\mathrm{TG}} = -1.4$ V 8

Supplementary Note 9: Conductance Replication in a Second Device 10

Supplementary Note 10: $B_{\perp}$-Dependence in Spectroscopy 10

Supplementary Note 11: Switching Current of the Planar SQUID 12

Supplementary Note 12: Adiabatic Theory of the Current–Phase Relation under Microwave Irradiation 15

Supplementary Note 13: Non-thermal ABS Occupation 16

Supplementary References 17

* fni@zurich.ibm.com

SUPPLEMENTARY NOTE 1: FREQUENCY DEPENDENCE OF CONDUCTANCE RESPONSE

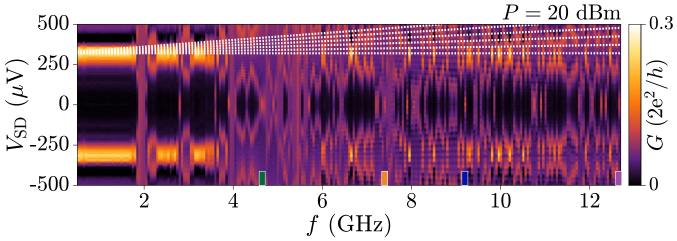

Supplementary Fig. 1. **Frequency dependence of conductance G as a function of source–drain bias V_{SD}, at a fixed microwave power $P = 20$ dBm.** Conductance replicas are schematically indicated by the dashed white lines, $\Delta V_{SD} = nhf/e$. Frequencies shown in Figs. 2 and 3 of the main text are indicated by coloured markers.

The microwave response of the device was first investigated as a function of microwave (MW) irradiation with frequency f, in the low barrier transparency regime ($V_T = -2.11$ V). Figure 1 shows the differential conductance G as a function of source–drain bias V_{SD} for increasing frequency from 500 MHz to 12.7 GHz, with an applied power $P = 20$ dBm. The conductance was unaffected by the applied signal for frequencies up to 1.8 GHz. At frequencies $f > 1.8$ GHz, the conductance was altered by the applied microwaves and in some cases we observed a non-zero conductance at $V_{SD} = 0$. The conductance response to irradiation frequency was non-monotonic, suggesting that the coupling strength of the antenna to the device was frequency-dependent. This was due to the method of applying microwaves by an exposed antenna within the sample space. The results shown in Figs. 2 and 3 of the Main Text were measured at frequencies labelled by the coloured markers. These frequencies were chosen where the response of the conductance was strongest based on Fig. 1, such that a full power dependence was possible.

Replication of conductance features, as those in the Main Text, were evident at both positive and negative bias. These followed a linear dependence on frequency, as highlighted by the white dashed lines at positive bias. Conductance replicas follow the relation $V_{SD} = nhf/e$, where n is an integer. Hence, the separation of conductance replicas of $\Delta V_{SD} = hf/e$ was consistent across a wide frequency range.

SUPPLEMENTARY NOTE 2: SPECTROSCOPY AT AN IN-PLANE MAGNETIC FIELD

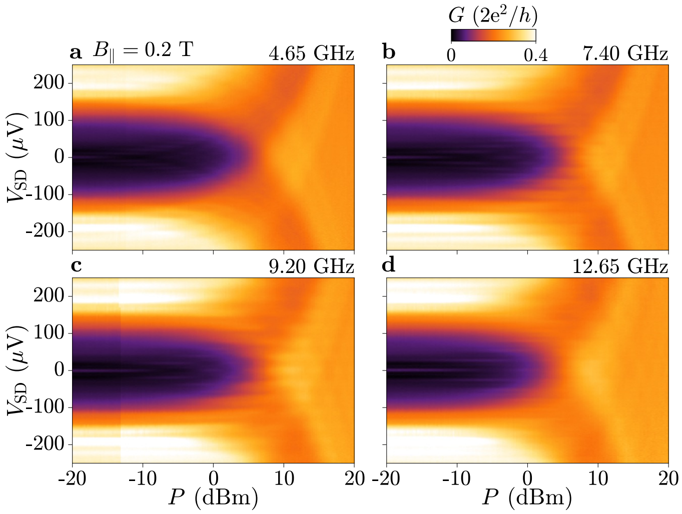

Supplementary Fig. 2. **Power dependence of conductance G as a function of bias V_{SD}, at an in-plane magnetic field $B_{\parallel} = 0.2$ T for different irradiation frequencies $f = \{4.65,\ 7.40,\ 9.20,\ 12.65\}$ GHz.**

In the Main Text, results were shown for an in-plane magnetic field $B_{\parallel} = 0$. Both the Josephson junction and the probe were in the superconducting state (S), meaning that tunnelling across the insulating barrier (I) corresponded

to an SIS geometry [as shown in Fig. 1(a) of the Main Text]. At an in-plane magnetic field of $B_{\parallel} = 0.2$ T, superconductivity in the probe was suppressed such that there was a finite density of states within the superconducting gap of the probe. The differential conductance G therefore showed features at bias values proportional to the density of states in the Josephson junction.

Figure 2 shows bias spectroscopy at an in-plane magnetic field of $B_{\parallel} = 0.2$ T as a function of power P, for different frequencies f of applied radiation. The device configuration was identical to the open regime outlined in the Main Text ($V_{\mathrm{T}} = -2.08$ V). The conductance G as a function of source–drain voltage V_{SD} shows a superconducting gap at low bias. Conductance values increased to a maximum close to $|V_{\mathrm{SD}}| = 200\ \mu$V. A small conductance peak was visible at $V_{\mathrm{SD}} = 0$, from a small residual supercurrent which flowed across the tunnel barrier.

On increasing microwave power P, replicas in conductance features emerged at both high and low bias. High-bias conductance replicas had separation $\Delta V_{\mathrm{SD}} = hf/\mathrm{e}$, as seen in the Main Text. Furthermore, the power dependence was similar to that at $B_{\parallel} = 0$. Conductance replicas were present under microwave irradiation when superconductivity was suppressed in the probe. This is consistent with photon assisted tunnelling (PAT) into Andreev bound states (ABSs) of charges in the probe at the Fermi energy.

SUPPLEMENTARY NOTE 3: MICROWAVE FIELD STRENGTH FROM SHAPIRO STEPS

The conductance of replicas appearing under microwave irradiation depends on the applied power P. We first considered the power dependence of Shapiro steps close to $V_{\mathrm{SD}} = 0$ [see blue dotted lines in Figs. 2(c) and 3(c) of the Main Text]. Conductance peaks occurred when the source–drain bias V_{SD} was equal to the Josephson voltage $V_{\mathrm{J}} = nhf/2\mathrm{e}$, where n is an integer denoting the order of the Shapiro step. The conductance of the nth Shapiro step is proportional to the nth-order Bessel function of the first kind, $J_n(2\mathrm{e}V_{\mathrm{MW}}/hf)$, where V_{MW} is the amplitude of the oscillating voltage due to the applied microwave signal. This corresponds to the most likely number of photons absorbed in the system. This scales linearly with n, such that there is an almost exact correspondence between V_{MW} and the V_{SD} at which the highest conductance peak occurs. The applied microwave signal is given as a power P in units of dBm. We therefore express the oscillating voltage at the sample as $V_{\mathrm{MW}} = V_0 \cdot 10^{P/20}$, where V_0 contains the output voltage, device–antenna coupling and coaxial line attenuation of 47 dB. In dimensionless units, the coupling strength to the microwave field is therefore defined as $\alpha \equiv \mathrm{e}V_{\mathrm{MW}}/hf = (\mathrm{e}/hf)V_0 \cdot 10^{P/20}$.

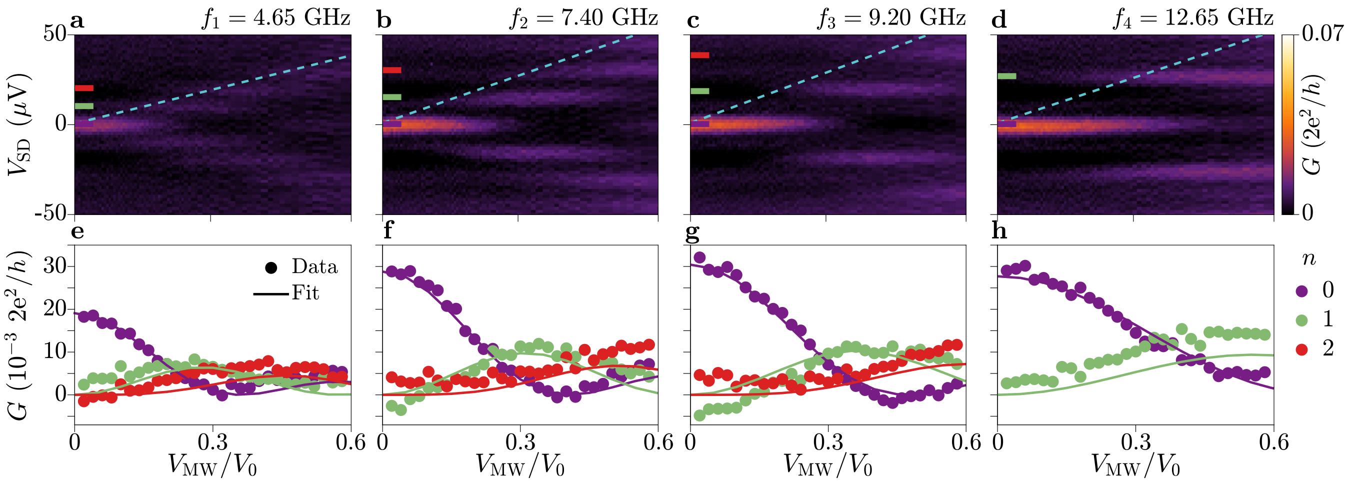

Supplementary Fig. 3. **Microwave field strength from Shapiro steps.** **(a–d)** Conductance of Figs. 2(b–e) of the Main Text, plotted as a function of microwave field strength $V_{\mathrm{MW}}/V_0 = 10^{P/20}$. Shapiro steps at $V_{\mathrm{SD}} = nhf/2\mathrm{e}$ are indicated by the coloured bars. Blue dashed lines indicate V_0, as calculated from a fit. **(e–h)** Linecuts of conductance in (a–d) at bias values corresponding to the $n = 0,\ 1,\ 2$ order Shapiro steps (circles). Fit to the conductance (lines) to obtain the parameter V_0.

The Shapiro steps in the closed regime [Figs. 2(b–e) of the Main Text] are plotted in Fig. 3(a–d) as a function of microwave field strength V_{MW}/V_0. The emergence of the nth Shapiro step scales linearly with V_{MW} as indicated by the blue dashed lines, the gradient of which is given by V_0. Linecuts of the zeroth, first and second order Shapiro steps are plotted in Figs. 3(e–h) as the purple, green and red circles, respectively. The plotted data is sampled from the raw data at intervals $\Delta V_{\mathrm{MW}}/V_0 = 0.02$, to have a regular separation of datapoints. The conductance of the nth Shapiro step, $G_n(V_{\mathrm{MW}}) \equiv G(V_{\mathrm{SD}} = nhf/2\mathrm{e},\ V_{\mathrm{MW}})$, is fitted with a squared Bessel function of the form [1–5]

$$G_n(V_{\rm MW}) = G_n(V_{\rm MW}=0)\left[J_n\left(\frac{2{\rm e}}{hf}V_{\rm MW}\right)\right]^2 = G_n(V_{\rm MW}=0)\left[J_n\left(\frac{2{\rm e}}{hf}V_0\cdot 10^{P/20}\right)\right]^2, \tag{S.1}$$

with $G_n(V_{\rm MW}=0)$ the conductance at bias $V_{\rm SD} = nhf/2{\rm e}$ with no microwaves applied. The fit with the free parameter V_0 returns $V_0 = \{64,\ 91,\ 96,\ 87\}$ μV and is plotted as the lines in Fig. 3(e–h) for frequencies f_1 to f_4, respectively. The corresponding dimensionless microwave field strengths are $\alpha_0 = \{3.3,\ 3.0,\ 2.5,\ 1.7\}$.

SUPPLEMENTARY NOTE 4: MODELLING OF PHOTON ASSISTED TUNNELLING DATA

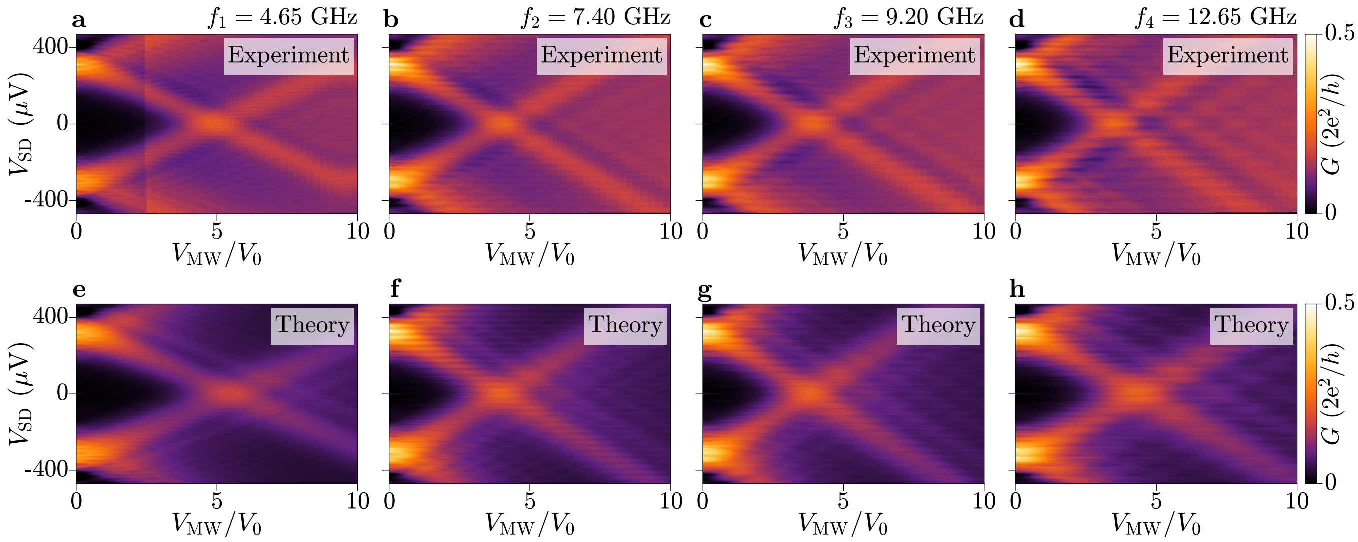

Supplementary Fig. 4. **Experimental and simulated conductance replicas as a function of microwave field strength. a–d** Differential conductance of Figs. 2(b–e) of the Main Text, plotted as a function of microwave field strength $V_{\rm MW}/V_0 = 10^{P/20}$. **(e–h)** Simulated conductance features as a function of microwave field strength, using the coupling parameters V_0 obtained in Fig. 3 and the measured conductance in the absence of microwave irradiation.

Figures 4(a–d) show the conductance maps of Figs. 2(b–e) of the Main Text plotted as a function of microwave field strength $V_{\rm MW}/V_0$. Conductance replicas emerge linearly with increasing microwave field strength. The experimental data is simulated using a model for photon assisted tunnelling, based on the coupling parameters V_0 obtained from the Shapiro steps [see Fig. 3]. The $n^{\rm th}$-order conductance replicas are expected to scale as a squared Bessel function [1–5]:

$$G_n\left(V_{\rm MW},\ V_{\rm SD}+n\frac{hf}{\rm e}\right) = G\left(V_{\rm MW}=0,\ V_{\rm SD}+n\frac{hf}{\rm e}\right)\left[J_n\left(\frac{{\rm e}V_{\rm MW}}{hf}\right)\right]^2. \tag{S.2}$$

Using the experimentally measured conductance with no applied microwaves, $G(V_{\rm MW}=0,\ V_{\rm SD})$, the conductance at each $V_{\rm MW}$ was calculated by summing the contributions from N replicas:

$$G(V_{\rm MW},\ V_{\rm SD}) = \sum_{n=-N}^{N} G_n\left(V_{\rm MW},\ V_{\rm SD}\right), \tag{S.3}$$

where $N = (1\ {\rm mV})\cdot {\rm e}/hf$ was chosen to consider conductance replicas emerging across the full range of measured source–drain bias. The simulated conductance is plotted in Figs. 4(e–h) as a function of microwave field strength $V_{\rm MW}/V_0$, using the values of V_0 obtained from the Shapiro steps at each frequency. The replication of conductance features is well described by the simulation, up to the highest measured microwave fields, in terms of the number of replicas, their dependence of microwave field strength and the absolute value of their conductance. Some discrepancy

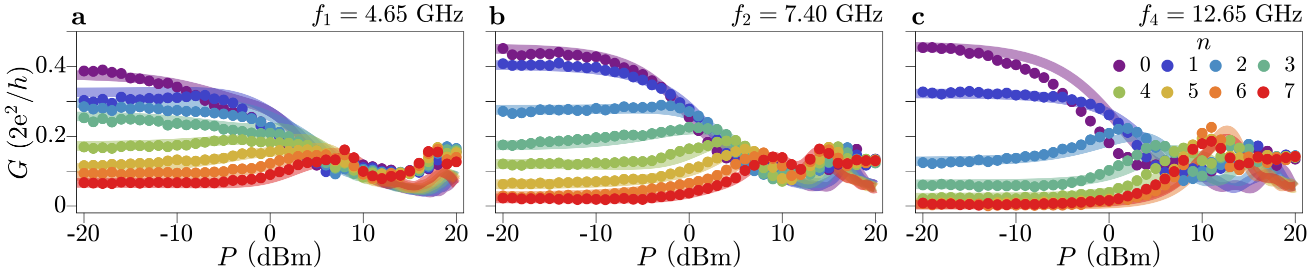

Supplementary Fig. 5. **Power dependence of conductance replicas.** Conductance of the first seven replica peaks in Fig. 2(b, c, e) of the Main Text (circles), respectively. Colours denote the order n of the replica. Plotted alongside simulated conductance from Fig. 4 (lines), as a function of applied microwave power P.

at large V_{MW}/V_0 can be attributed to a background conductance in the measurement data, potentially due to device heating which is not accounted for in simulation.

Figure 5 shows the conductance of replica peaks at fixed bias V_{SD} as a function of applied power P, up to the seventh replica [circles, replica number indicated by the colour]. Data is plotted for frequencies $f_1 = 4.65$ GHz, $f_2 = 7.40$ GHz and $f_4 = 12.65$ GHz, since the equivalent data for $f_3 = 9.20$ GHz is plotted in Fig. 4(c) of the Main Text. The simulated conductance at the same bias is plotted as the shaded lines, and matches the data for low and intermediate powers $P \lesssim 10$ dBm. Data is plotted for $P \gtrsim -20$ dBm to better highlight the power dependence, since only small changes in conductance were observed in the range -40 dBm $< P \lesssim -20$ dBm.

SUPPLEMENTARY NOTE 5: REMOVAL OF BACKGROUND CONDUCTANCE

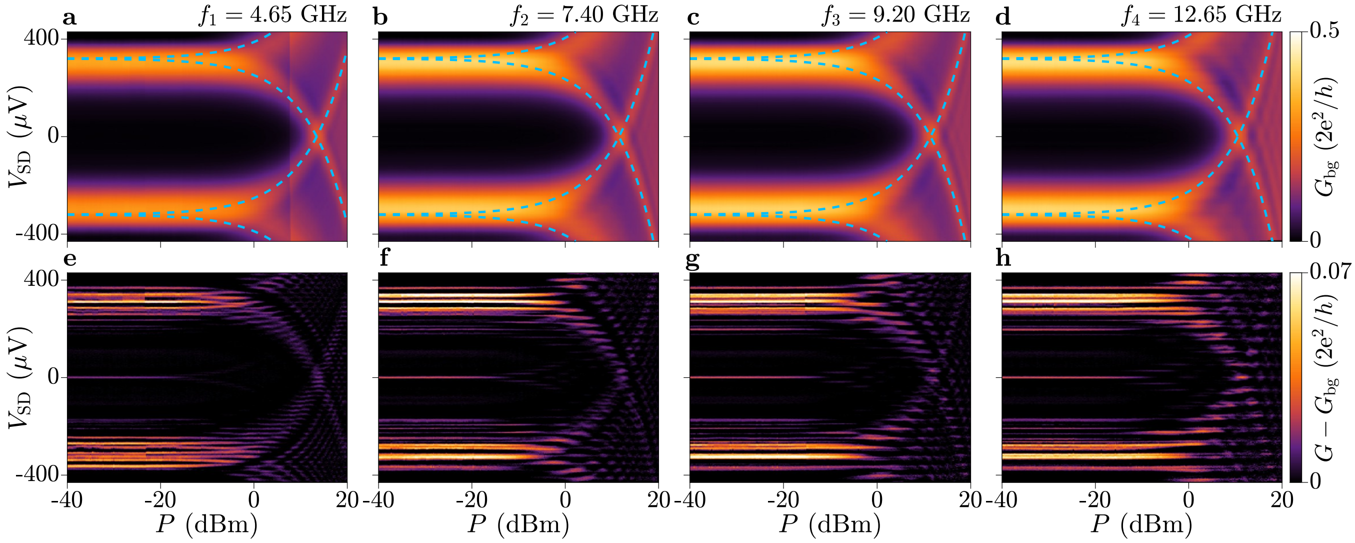

Supplementary Fig. 6. **Bias spectroscopy with the removal of a slowly-varying background (a–d)** Power dependence of Fig. 2 in the Main Text at different frequencies, averaged at each power across a bias voltage window $V_{\mathrm{window}} = 70$ μV. Dashed lines indicate the power dependence of high conductance features. **(e–h)** Power dependence of Fig. 2 in Main Text, with the averaged background removed $G - G_{\mathrm{bg}}$. Linecuts in Fig. 3(a) of the Main Text are taken at powers $P = \{1.5,\ 4.5,\ 4,\ 4.5\}$ dBm, respectively.

The conductance maps in Fig. 2 of the Main Text show the complete response of the system to a microwave drive of increasing power. Figures 6(a–d) show a slowly-varying background conductance G_{bg}, obtained by averaging the conductance trace at each power P over a bias window of 70 μV. Dashed lines show the dependence of high conductance features on power P, with the relation $V_{\mathrm{MW}} = V_0 \cdot 10^{P/20}$ for values of V_0 calculated from the Shapiro steps [see Fig. 3].

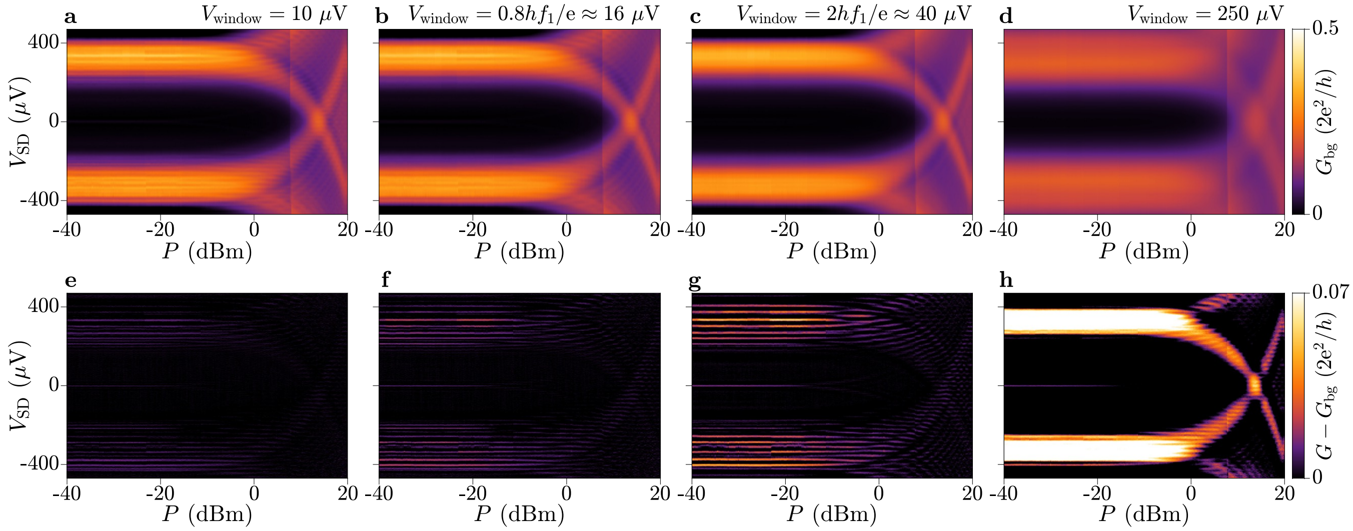

Supplementary Fig. 7. **Dependence of background conductance removal on averaging window size for small microwave frequency (a–d)** Power dependence of Fig. 2(b) in the Main Text, frequency $f = 4.65$ GHz, averaged at each power across different sizes of bias voltage window V_{window}. **(e–h)** Power dependence of Fig. 2(b) in Main Text, with the averaged background removed $G - G_{\text{bg}}$.

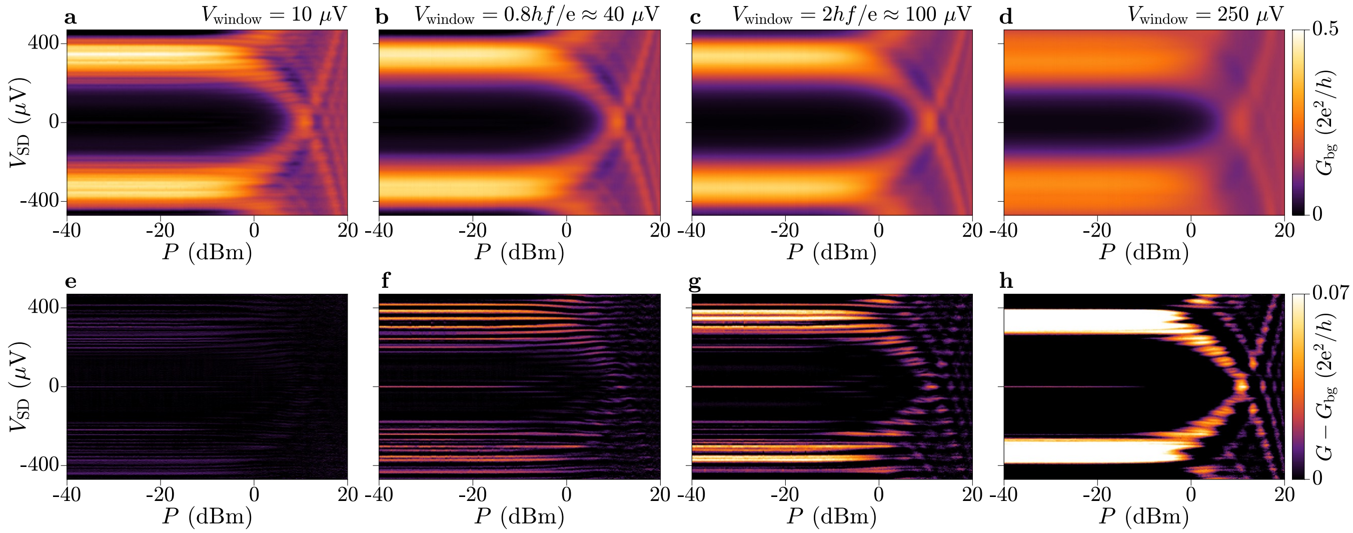

Supplementary Fig. 8. **Dependence of background conductance removal on averaging window size for small microwave frequency** Same as Fig. 7, for the data in Fig. 2(e) of the Main Text, corresponding to a frequency $f = 12.65$ GHz.

Similar plots to Fig. 6 are shown in Figs. 7 and 8, which present the data at $f = 4.65$ GHz and $f = 12.65$ GHz, respectively, for different sizes of bias window. For a small bias window, $V_{\text{window}} \lesssim hf/\text{e}$ [Figs. 7(a, b) and 8(a, b)], conductance replicas are included in G_{bg} and so are not fully separated when plotting $G - G_{\text{bg}}$. For a large bias window, $V_{\text{window}} \gtrsim 250\ \mu$V [Figs. 7(d) and 8(d)], the bias dependence of the conductance background G_{bg} is averaged over a large range, meaning that features are smeared and not representative of the device. The bias window was therefore chosen to be larger than the maximum voltage separation of conductance replicas, corresponding to $\sim 50\ \mu$V for $f = 12.65$ GHz. A bias window of 70 μV fulfilled this criteria for all frequencies, while being sufficiently small as to limit distortions to the background conductance G_{bg}.

Conductance replicas were isolated by subtracting the slowly-varying background, $G - G_{\text{bg}}$ [see Figs. 6(e–h)]. The linecuts in Fig. 4(a) of the Main Text were taken at powers $P = 1.5$ dBm, 4.5 dBm, 4 dBm and 4.5 dBm from Figs. 6(e–h) respectively, such that multiple conductance replicas were visible. The separation between conductance features ΔV_{SD} shown in Fig. 4(b) of the Main Text was calculated by taking the average of conductance peak separation

across the full power range displayed in Figs. 6(e–h). Conductance replicas at low bias [squares in Fig. 4(b) of the Main Text] were calculated in a similar way, using replicas across the full range of power in both the tunnelling regime [Fig. 2 of the Main Text] and the more open barrier regime [Fig. 3 of the Main Text].

The background conductance contained features from the complex ABS spectrum at low power. Multiple high-conductance lines were visible, in both the background conductance and the difference, due to replication of different features in the low-power conductance map. Such conductance features could include sub-gap ABSs at $V_{\rm SD} = \pm(\Delta + E_{\rm A})/e$ and the superconducting gap at $V_{\rm SD} = 2\Delta/e$.

SUPPLEMENTARY NOTE 6: SUM RULE FOR CONDUCTANCE REPLICAS

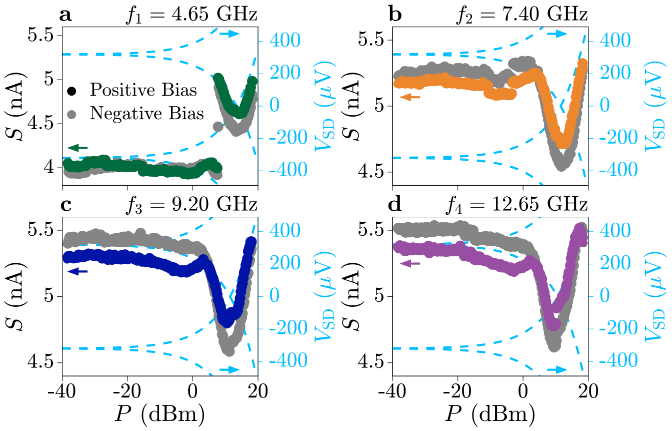

Supplementary Fig. 9. **Sum of conductance replicas under microwave irradiation of different frequencies. (a–d)** (Left axis) Sum of conductance over bias, S, as a function of power P for the data in Figs. 2(b–e) of the Main Text, respectively. Frequencies f_i correspond to those of Fig. 2 in the Main Text, where colours are defined. Coloured (grey) circles correspond to a sum over positive (negative) bias, $V_{\rm SD} > 0$ ($V_{\rm SD} < 0$). (Right axis) Dashed lines indicate the power dependence of conductance replicas, identical to Fig. 6.

Reference [6] described the importance of a sum rule for conductance replicas to support their interpretation of Floquet–Andreev (F–A) states emerging under microwave irradiation. The sum rule brought forward in Ref. [6] states that the sum of conductance over source-drain bias should be constant as a function of power, independent of the emergence of conductance replicas. This is expressed by the equation $S = \int_0^{\pm\infty} ({\rm d}I/{\rm d}V){\rm d}V$, which is equivalent to a numerical integral of the experimental data. We applied the same technique to the results shown in Figs. 2(b–e) of the Main Text [see Fig. 9, data of Fig. 9(c) also plotted in inset of Fig. 4(d) of the Main Text]. The sum S was calculated for each value of applied power P by numerical integration of the differential conductance $G \equiv {\rm d}I/{\rm d}V$ over positive (negative) bias values, indicated by the coloured (grey) circles (left axis). Dashed lines in Fig. 9 indicate the power dependence of high conductance features, as a function of bias $V_{\rm SD}$ (right axis). The power dependence is identical to those shown in Fig. 6(a–d). The sum S was approximately constant as a function of power up to $P \approx 5$ dBm. For $P \gtrapprox 5$ dBm, high conductance features were outside of the measurement range $-490\ \mu$V $< V_{\rm SD} < 490\ \mu$V. The change in S was therefore consistent with conductance replicas exiting the measurement range, such that they were not included in S. The constant S at low power is consistent with our conclusion that PAT was the dominant mechanism for conductance replicas, since it represents conservation of the number of states in the tunnel barrier and the junction. Equivalently, using Eq. S.2 we see that $S \propto \sum_n J_n^2(x)$ which is constant for a sum over all n. Hence, the total tunnel current through the barrier is constant as a function of power.

SUPPLEMENTARY NOTE 7: MICROWAVE COUPLING STRENGTH FROM HIGH-BIAS CONDUCTANCE

The coupling strength to the microwave field was calculated from the Shapiro steps in Fig. 3. We complement these values with calculations of the coupling strength directly from conductance features at high source–drain bias $V_{\rm SD}$. First, the background conductance [see Figs. 6(a–d)] is fitted with a Gaussian function for each value of power P, or equivalently each value of microwave field amplitude $V_{\rm MW}$. Thus, values for the conductance peak position $V_{\rm p}(V_{\rm MW})$

and standard deviation $\sigma(V_{\rm MW})$ are obtained as a function of $V_{\rm MW}$. Then, the conductance peak position is fitted with a linear curve to obtain V_0. The values $V_{\rm p}(V_{\rm MW})$ included in the fit are weighted by the standard deviations $\sigma(V_{\rm MW})$. This method produces a value of V_0 for each microwave frequency f, along with an error δV_0 describing the uncertainty of the coupling strength to describe the data given the standard deviation σ. The obtained values are $V_0 \pm \delta V_0 = \{83.7{\pm}0.4,\ 97.6{\pm}1.5,\ 99.5{\pm}1.7,\ 100.6{\pm}2.9\}$ μV, for frequencies $f = \{4.65,\ 7.20,\ 9.40,\ 12.65\}$ GHz respectively. The corresponding values of the dimensionless coupling strength are $\alpha_0 = \{4.35{\pm}0.02,\ 3.19{\pm}0.05,\ 2.62{\pm}0.04,\ 1.92{\pm}0.06\}$. The standard deviation of the conductance peak was $60 < \sigma < 75$ μV for all datasets.

SUPPLEMENTARY NOTE 8: RESULTS FOR $V_{\rm TG} = -1.4$ V

Conductance replication demonstrated in the Main Text was obtained at $V_{\rm TG} = -0.8$ V. Here we show measurements on the same device at $V_{\rm TG} = -1.4$ V. Figure 10 shows the differential conductance G as a function of bias $V_{\rm SD}$ and tunnel gate voltage $V_{\rm T}$, when the top-gate voltage was set to $V_{\rm TG} = -1.4$ V. For high tunnel barrier transparency, conductance features at $V_{\rm SD} = 0$ indicate remnants of a supercurrent between the probe and the SNS junction. Regularly spaced finite-bias features are consistent with multiple Andreev reflections. For low tunnel barrier transparency, measurements are in the tunnelling regime and the conductance is indicative of the density of states in the SNS junction.

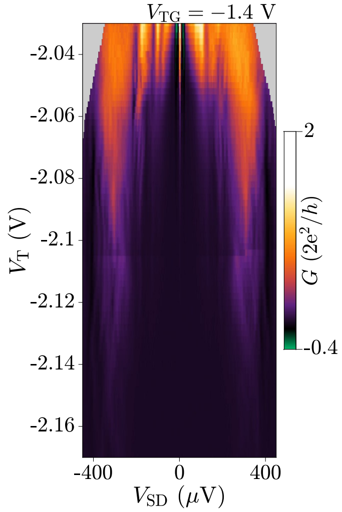

Supplementary Fig. 10. **Tunnel barrier dependence at more negative gate voltage.** Differential conductance G as a function of bias $V_{\rm SD}$ and tunnel barrier gate voltage $V_{\rm T}$, at a top-gate voltage $V_{\rm TG} = -1.4$ V.

Figures 12–15 show bias-spectroscopy as a function of applied microwave power, for tunnel gate voltages $V_{\rm T} = -2.06$ V, -2.08 V, -2.1 V and -2.12 V respectively. For high tunnel barrier transparency [$V_{\rm TG} = -2.06$ V, Fig. 12], a conductance peak at $V_{\rm SD} = 0$ was indicative of a supercurrent flowing across the tunnel barrier. On increasing applied microwave power, conductance replicas emerged in both the low and high bias features, at the same power and with the same dependence. For lower tunnel barrier transparency [Figs. 13–15], conductance features at high bias were replicated with separation $\Delta V_{\rm SD} = hf/{\rm e}$. The mean separation of low and high bias replicas are displayed as filled grey squares and circles in Fig. 4(b) of the Main Text, respectively. The bias separation of conductance replicas was consistent with $\Delta V_{\rm SD} = hf/q$, where q is the charge tunnelling across the barrier. As in the Main Text, concurrent replicas in low and high bias features indicated PAT as the dominant mechanism.

The coupling strength to the microwave field is calculated for this $V_{\rm TG}$ value from conductance features at high source–drain bias in Fig. 14, using the same procedure as outlined in the previous section. The obtained values are $V_0 \pm \delta V_0 = \{80.6 \pm 2.3,\ 100.2 \pm 2.6,\ 99.1 \pm 2.7,\ 96.0 \pm 2.3\}$ μV, for frequencies $f = \{4.65,\ 7.20,\ 9.40,\ 12.65\}$ GHz respectively. The corresponding values of the dimensionless coupling strength are $\alpha_0 = \{4.19{\pm}0.12,\ 3.27{\pm}0.08,\ 2.60{\pm}0.07,\ 1.84 \pm 0.04\}$. As for the data taken at $V_{\rm TG} = -0.8$ V, the standard deviation of the conductance peak was $60 < \sigma < 75$ μV for all datasets.

The values for α_0 at $V_{\rm TG} = -1.4$ V show remarkable agreement with those at -0.8 V [see blue lines in Figs. 14(c–f)]. The change in coupling strength as a result of the more negative $V_{\rm TG}$ is quantified by $\Delta\alpha_0 \equiv \alpha_0(V_{\rm TG} = -0.8) - \alpha_0(V_{\rm TG} = -1.4) = \{-0.16 \pm 0.12,\ 0.08 \pm 0.09,\ -0.02 \pm 0.08,\ 0.08 \pm 0.07\}$ for the respective frequencies of

applied microwave radiation. Uncertainties are calculated from the sum over variances of each α_0 value. These results show that the change in coupling strength as a result of the more negative gate voltage was at most 4%.

The carrier density in the SNS junction is expected to change as a function of V_{TG}. The maximum switching current I_0 of Device 1 is plotted in Fig. 11(a) as a function of V_{TG}. Data points corresponding to $V_{\mathrm{TG}} = -0.8$ V and -1.4 V are indicated by dashed lines, and show that I_0 at $V_{\mathrm{TG}} = -1.4$ V was $\sim$ 25% the value at $V_{\mathrm{TG}} = -0.8$ V. The change in maximum switching current was $\Delta I_0 \approx 0.8$ μA. At $V_{\mathrm{TG}} = -0.8$ V, I_0 reached a peak after a linear increase from the most negative V_{TG} values. We associate this linear regime to occupation of the first subband in the semiconductor. The open ($V_{\mathrm{TG}} = -0.8$ V) and closed ($V_{\mathrm{TG}} = -1.4$ V) regimes are therefore associated with full and partial occupation of the first subband, respectively. Gated Hall bar measurements in the same material are shown in Fig. 11(b). The density n and mobility μ are plotted as a function of the gate voltage V_{G}. The gate lever arm was different in the Hall bar and SNS junction due to different fabrication processes for each chip. Therefore, we estimate the change in density from the range of single subband occupation, where the mobility μ increased linearly with n. The carrier density at peak μ was compared to that where the mobility was 25% above its lowest measured value. This was chosen to approximately correspond to the I_0 value at $V_{\mathrm{TG}} = -1.4$ V relative to $V_{\mathrm{TG}} = -0.8$ V. This gave an approximate change in carrier density of $\Delta n \approx 0.5 \cdot 10^{12}$ cm^{-2}, or $\Delta n/n \approx 0.5$. We therefore estimate a 25% decrease in the Fermi velocity for $V_{\mathrm{TG}} = -1.4$ V relative to $V_{\mathrm{TG}} = -0.8$ V. While this value is an approximation, the large change in I_0 is indicative of an appreciable change in the carrier density.

From the theory of Floquet–Andreev states [6], a 25% decrease in the Fermi velocity would correspond to a 25% decrease in the microwave coupling strength α_0. This is plotted as the yellow lines in Figs. 14(c–f), and does not match the experimental result. To be consistent with a Floquet–Andreev interpretation, calculated values of $\Delta\alpha_0$ imply a change in the Fermi velocity of less that 4%, incompatible with switching current and Hall bar measurements, or an alternative mechanism which almost exactly compensates for the change in carrier density. In contrast, no gate dependence is expected in the PAT interpretation. This further supports PAT as the dominant mechanism for conductance replicas.

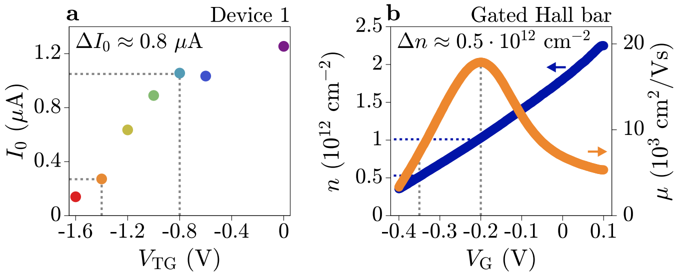

Supplementary Fig. 11. **Estimating the change in carrier density as a function of V_{TG}.** (a) Maximum switching current I_0 in Device 1 as a function of top-gate voltage V_{TG}. Data points at $V_{\mathrm{TG}} = -0.8$ V and -1.4 V are indicated by dashed lines, corresponding to a difference $\Delta I_0 \approx 0.8$ μA. (b) Measurements of a gated Hall bar in the same material. Carrier density n (blue, left axis) and mobility μ (orange, right axis) are plotted as a function of the global gate voltage V_{G}. Dashed lines indicate the estimated change in carrier density as $\Delta n \approx 0.5 \cdot 10^{12}$ cm^{-2}.

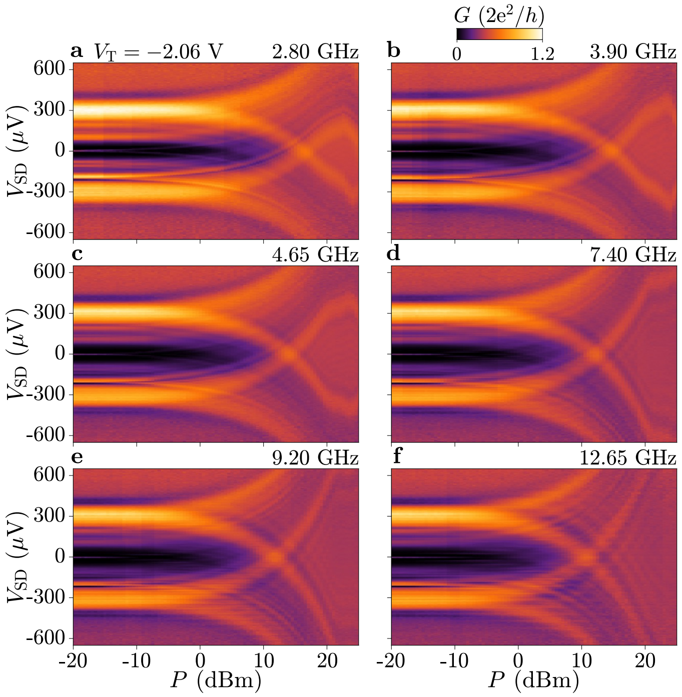

Supplementary Fig. 12. **Power dependence at** $V_{\mathrm{TG}} = -1.4$ V **and** $V_{\mathrm{T}} = -2.06$ V**. (a–f)** Conductance G as a function of source–drain bias V_{SD} and power P, for frequencies $f = \{2.80,\ 3.90,\ 4.65,\ 7.40,\ 9.20,\ 12.65\}$ GHz. Mean separation of replicated supercurrent features is shown in Fig. 4(b) of the Main Text (full grey squares).

SUPPLEMENTARY NOTE 9: CONDUCTANCE REPLICATION IN A SECOND DEVICE

Measurements were performed on a second device, fabricated on the same chip and lithographically similar to the first except for the width of the SNS junction, which was 500 nm rather than 2.5 μm for Device 1. Measurements are shown for tunnel gate voltages $V_{\mathrm{T}} = -0.768$ V a top gate voltage $V_{\mathrm{TG}} = 0$ V, kept constant throughout the measurements. Figure 16 shows the frequency response of Device 2 to microwave irradiation at an applied power of $P =$ 20 dBm. The frequency response was similar to that of Device 1 [see Fig. 1], showing conductance replicas with separation $\Delta V_{\mathrm{SD}} = hf/\mathrm{e}$ indicated by the white dashed lines. Frequencies $f = 4.70$ GHz, 6.85 GHz, 9.45 GHz and 11.90 GHz are indicated by the coloured markers, where many replicas are evident. Figure 17 shows the conductance response to microwave irradiation at these frequencies, for increasing microwave power P. Conductance replicas emerged with separation $\Delta V_{\mathrm{SD}} = hf/\mathrm{e}$, shown as empty grey circles in Fig. 4(b) of the Main Text. Figure 18 shows the conductance as a function of perpendicular magnetic field $B_{\perp}$, for increasing microwave power. Field-periodic conductance features were replicated, with more replicas emerging for increasing applied power.

Figure 19 shows the differential conductance as a function of applied power when the transparency of the tunnel barrier was significantly reduced, by setting tunnel gate voltages to $(V_{\mathrm{T,L}},\ V_{\mathrm{T,R}}) = (-0.911,\ -0.875)$ V. Conductance replicas emerge up to large applied powers, as in Fig. 17.

SUPPLEMENTARY NOTE 10: $B_{\perp}$-DEPENDENCE IN SPECTROSCOPY

Selected conductance maps as a function of perpendicular magnetic field $B_{\perp}$ are shown in Figs. 5(a–c) of the Main Text. The full dataset is shown in Fig. 20, for no applied microwaves [Fig. 20(a)] and applied powers ranging from $P = -10$ dBm [Fig. 20(b)] to $P = 10$ dBm [Fig. 20(f)]. Some conductance features were periodic in $B_{\perp}$; these corresponded to ABSs in the SNS junction, which were dependent on the phase difference across the junction. Field-independent features corresponded to the superconducting gap edge at $V_{\mathrm{SD}} = 2\Delta/\mathrm{e}$, and conductance resonances in the tunnelling probe. For increasing microwave power, additional field-periodic features appeared in the conductance map. The magnitude of the conductance at a given bias decreased, as it was distributed across more conductance peaks. This is consistent with current conservation in the PAT process.

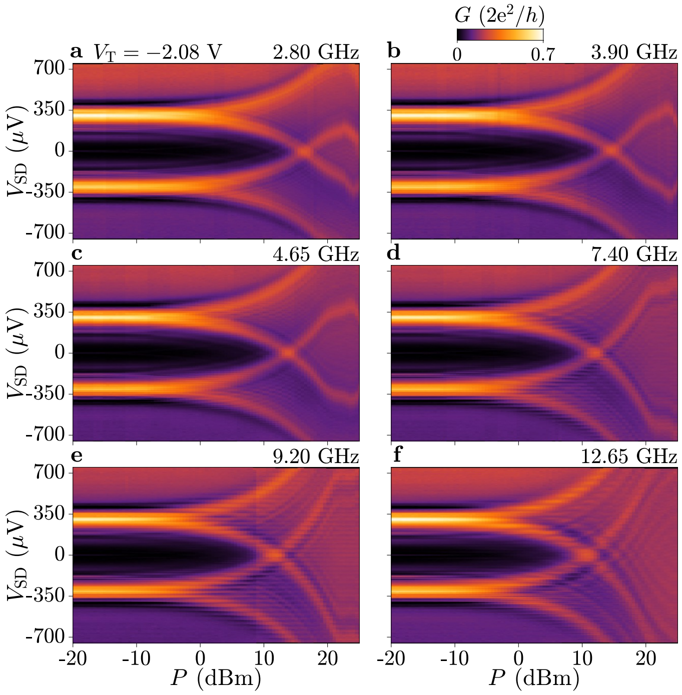

Supplementary Fig. 13. Same as 12 for $V_T = -2.08$ V.

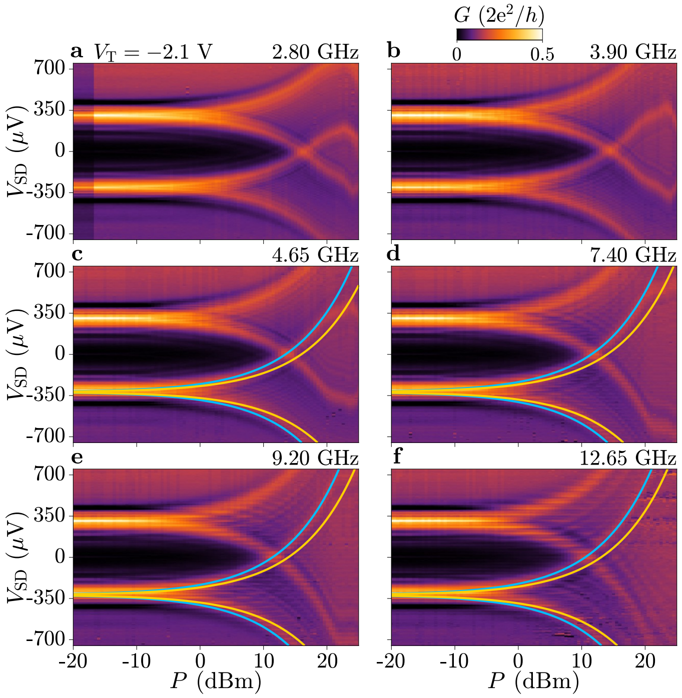

Supplementary Fig. 14. Same as 12 for $V_T = -2.1$ V. Mean separation of replicated conductance features is shown in the Fig. 4(b) of the Main Text (full grey circles). Power dependence of conductance replicas obtained for $V_{TG} = -0.8$ V [blue lines, identical to Fig. 6] is plotted in (c–f), alongside the expectation for a 25% decrease in coupling strength due to smaller Fermi velocity [yellow lines].

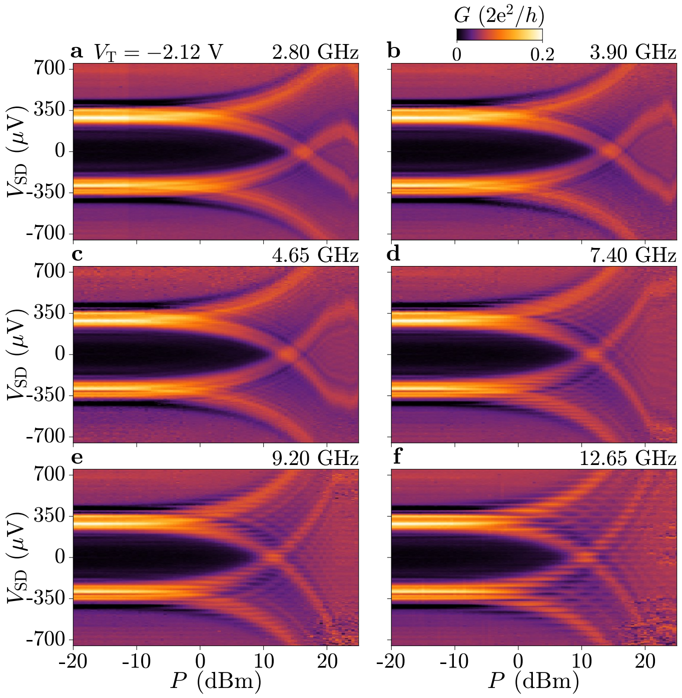

Supplementary Fig. 15. Same as 12 for $V_T = -2.12$ V.

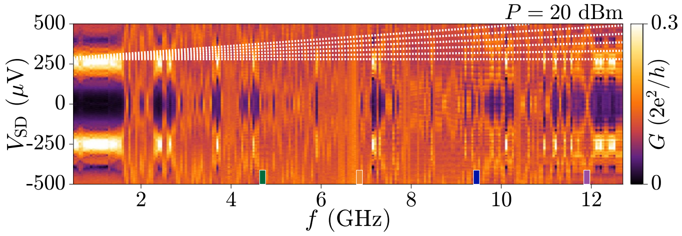

Supplementary Fig. 16. **Frequency dependence of conductance G as a function of source–drain bias V_{SD} in Device 2, at fixed power $P = 20$ dBm.** Conductance replicas are schematically indicated by the dashed white line, $\Delta V_{SD} = hf/\mathrm{e}$. Coloured markers indicate the frequencies used in Figs. 17 and 18.

SUPPLEMENTARY NOTE 11: SWITCHING CURRENT OF THE PLANAR SQUID

Current-biased measurements were performed by applying a current I_{DC} to a low-impedance superconducting lead on the right side of the planar SQUID loop, which flows to ground via a second low-impedance superconducting lead at the bottom of the device [see Fig. 1(c) of Main Text]. The current was prevented from flowing through the probe by floating its contacts. The differential voltage drop across the planar SQUID, V_2, was measured to detect the switching current to the resistive state. The SNS junction was embedded in a superconducting loop defined by a 400 nm wide epitaxial Al stripe enclosing an area of $A = 10\ \mu\mathrm{m}^2$. The width of a 200 nm portion of the loop was reduced to 130 nm, which reduced the switching current of the loop from several mA to 36 μA. Without this Al constriction, the switching current background would be too large to be measured without dissipating large amounts of heat at the mixing chamber of the fridge. The switching current of the Al constriction was still more than a factor of 30 larger than the switching current of the SNS junction. Due to the large asymmetry in the critical currents of the planar SQUID, the oscillations correspond to the current–phase relation (CPR) of the SNS junction and the background to the Al constriction. Hence, a perpendicular magnetic field $B_\perp$ applied to the loop of area A resulted in a phase drop of $\varphi = 2\pi B_\perp A/\Phi_0$ across the SNS junction.

The switching current of the planar SQUID is shown in Fig. 21(a), for increasing microwave power P. At low power [purple circles], oscillations with a period of $B_{\mathrm{Period}} \approx 200\ \mu$T and peak-to-peak amplitude of 2 μA were observed, on top of a constant background of 36 μA. For increasing applied power, the amplitude of the oscillations decreased

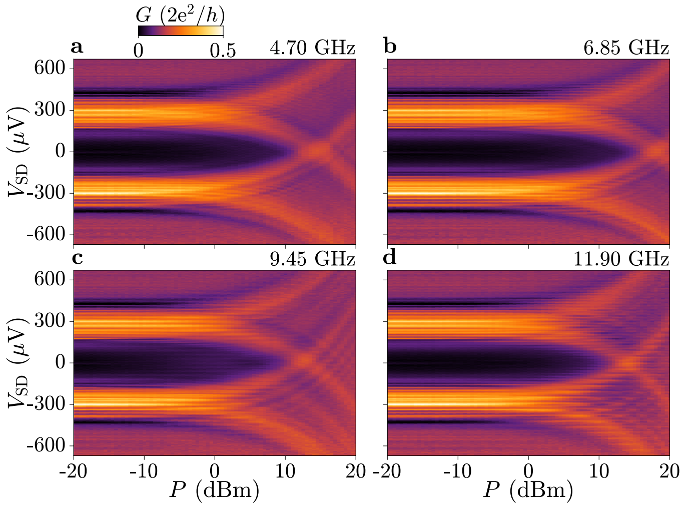

Supplementary Fig. 17. **Conductance of Device 2 as a function of applied microwave power P, for frequencies** $f = \{4.70,\ 6.85,\ 9.45,\ 11.90\}$ GHz **in (a–d) respectively.**

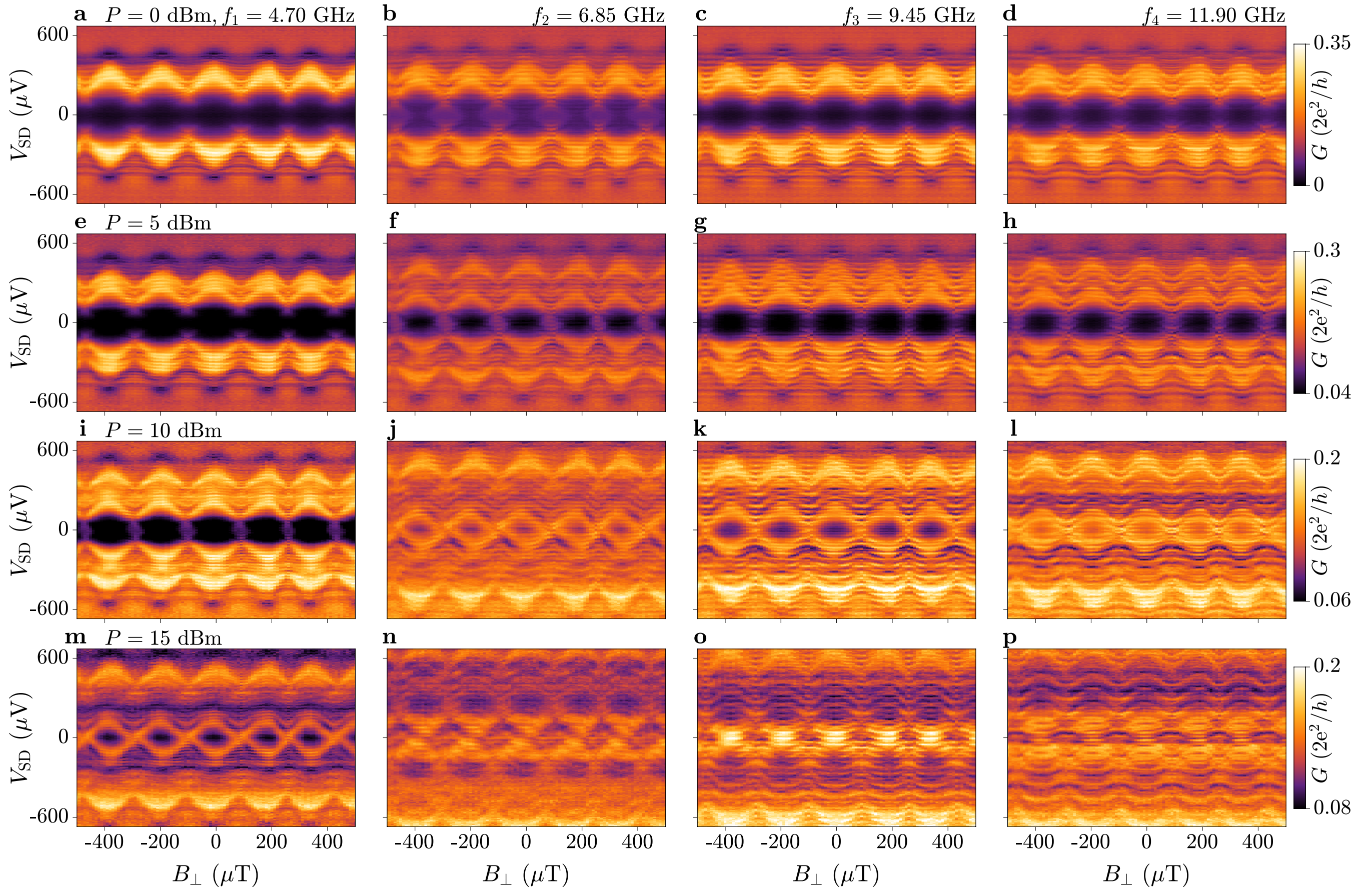

Supplementary Fig. 18. **Conductance of Device 2 as a function of perpendicular magnetic field $B_\perp$ for different frequencies f and powers P.** **(a–d)** $B_\perp$ dependence of conductance response at $P = 0$ dBm for frequencies $f = \{4.70,\ 6.85,\ 9.45,\ 11.90\}$ GHz, respectively. **(e–h)** Same as (a–d) for $P = 5$ dBm. **(i–l)** Same as (a–d) for $P = 10$ dBm. **(m–p)** Same as (a–d) for $P = 15$ dBm.

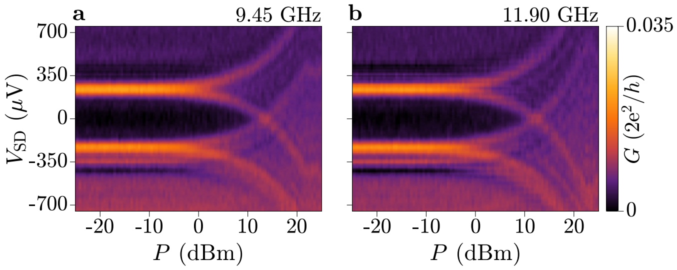

Supplementary Fig. 19. **Conductance replicas in Device 2 at low tunnel-barrier transparency** $(V_{\mathrm{T,L}},\ V_{\mathrm{T,R}}) = (-0.911,\ -0.875)$ V**, for frequencies** 9.45 GHz **and** 11.90 GHz **in (a, b) respectively.**

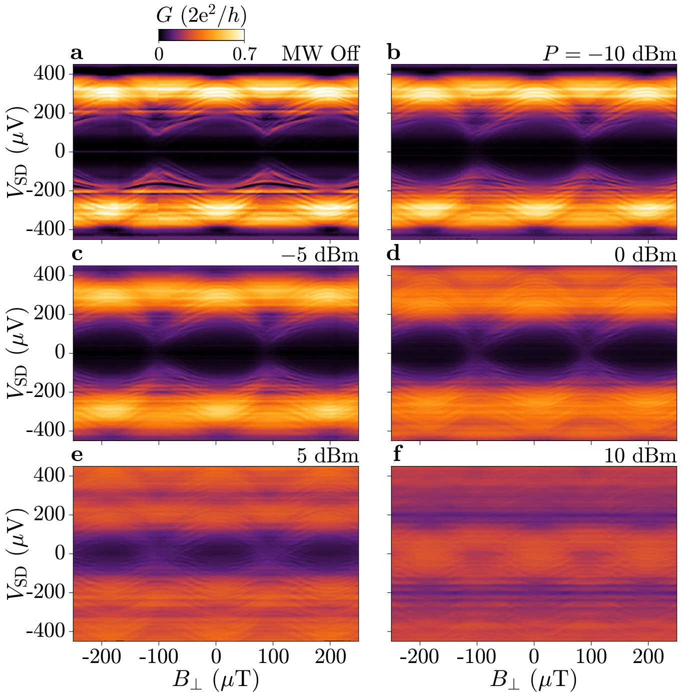

Supplementary Fig. 20. **Conductance as a function of perpendicular magnetic field** $B_\perp$**. (a)** Conductance G as a function of $B_\perp$ with no microwave field applied. **(b–f)** Conductance G as a function of $B_\perp$ under microwave irradiation at frequency $f = 9.20$ GHz. Applied microwave powers of $P = \{-10,\ -5,\ 0,\ 5,\ 10\}$ dBm, respectively. Same gate configuration as Fig. 4 in the Main Text.

and their shape was distorted (as described in the Main Text), while the background switching current decreased and developed a pronounced minimum close to $B_\perp = 0$. The decrease in the switching current of the constriction under microwave irradiation is assigned to pair-breaking in the Al by photon absorption, which may also account for the enhanced switching current suppression close to $B_\perp = 0$ by quasiparticle generation in the constriction and the superconducting leads [7].

The CPR of the SNS junction was obtained by subtracting the switching current of the constriction, as shown in Fig. 21(b) for $P = -40$ dBm [purple circles]. The switching current of the constriction was found by a polynomial fit to the data across six full periods, such that the resulting CPR was symmetric with respect to current and had a constant oscillation amplitude over all periods. The microwave field did not affect the switching current at this low power, so the CPR is considered to be at equilibrium. We described the data by extracting the harmonics up to the 10th order, using the following equation:

$$I_{\mathrm{eq}} = \sum_n I_n \sin(n\varphi), \tag{S.4}$$

where $I_n = (1/\pi)\int_0^{2\pi} I_{\mathrm{eq}} \sin(n\varphi)\mathrm{d}\varphi$.

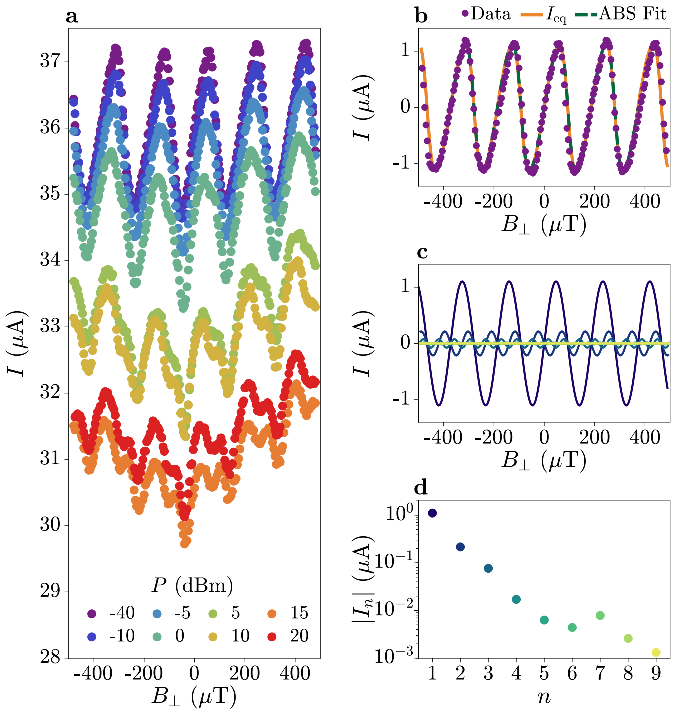

Supplementary Fig. 21. **Current phase relation under microwave irradiation at $f = 9.20$ GHz. (a)** Switching current I of the planar SQUID as a function of $B_\perp$, as a function of power P. **(b)** CPR data at $P = -40$ dBm, after subtraction of background current corresponding to Al constriction (circles). Equilibrium current I_{eq} determined from harmonics I_n up to tenth order (orange line). Fit to CPR using Eq. S.5 (green dashed line), giving an effective transmission of $\bar{\tau} = 0.84$. **(c)** Harmonics $I_n \sin(n\varphi)$ extracted from the low power CPR up to the tenth order. Colours are defined in (d). **(d)** Absolute amplitude $|I_n|$ of the harmonics plotted in (c).

The equilibrium supercurrent is plotted as the orange line in Fig. 21(b), composed of the harmonics in Fig. 21(c) with amplitudes $|I_n|$ plotted in Fig. 21(d). The presence of $n > 1$ terms, which gives the forward skewness of the CPR, is indicative of the presence of highly transmissive ABSs in the junction [8–10]. Since these ABSs carry the supercurrent, the CPR is described in terms of the ABS properties. However, the junction contained many modes, each with a distinct transmission τ, which all contribute to the supercurrent. It was not feasible to assign a transparency to each individual mode, so we instead considered a junction where all modes have an equal effective transmission $\bar{\tau}$. This describes the macroscopic properties of the junction, but does not capture details of the individual microscopic states. The CPR was then described by

$$I_{\mathrm{ABS}} = I_0 \frac{\bar{\tau}\sin(\varphi)}{E_{\mathrm{A}}(\varphi)/\Delta}, \tag{S.5}$$

where $E_{\mathrm{A}} = \Delta\sqrt{1 - \bar{\tau}\sin^2(\varphi/2)}$ is the ABS energy and $I_0 = (\mathrm{e}/2\hbar)\bar{N}\Delta$, where $\bar{N}$ is the effective number of modes in the junction. A fit to the low power data gave $\bar{\tau} = 0.84$ [green dashed line in Fig. 21(b)], consistent with the presence of highly transmissive modes observed in tunnelling spectroscopy (see Fig. 1(e) in the Main Text).

SUPPLEMENTARY NOTE 12: ADIABATIC THEORY OF THE CURRENT–PHASE RELATION UNDER MICROWAVE IRRADIATION

We use an adiabatic theory of an SNS junction under microwave irradiation to describe the CPR under increasing applied power [11–13]. A monochromatic drive at frequency f generates a time-varying voltage $V(t) = V_{\mathrm{MW}}\sin(2\pi f t)$, resulting in a time-varying phase across the SNS junction of $\varphi(t) = \varphi_0 + 2\alpha\cos(2\pi f t)$. The electromagnetic field strength is described by the parameter $\alpha = \mathrm{e}V_{\mathrm{MW}}/hf$. In the adiabatic approximation, the stationary phase at equilibrium [Eq. S.4] is replaced by the time-varying phase $\varphi(t)$. No excitation of ABSs is considered in this model. The resulting CPR is:

$$I_{\text{ad.}} = \sum_n I_n J_0(2n\alpha)\sin(n\varphi), \quad \text{(S.6)}$$

where J_0 is a zero-order Bessel function of the first kind and I_n are the harmonic coefficients obtained for the equilibrium CPR. The CPR traces under microwave irradiation were therefore fitted with α as a single free parameter, using the I_n shown in Fig. 21(d). The results of the fit are shown in Fig. 5(e) of the Main Text.

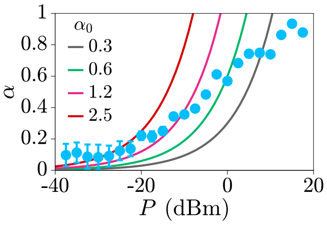

Supplementary Fig. 22. **Power dependence of microwave coupling strength** Microwave coupling strength α, obtained from a fit the data in Fig. 5(d) of the Main Text, as a function of power P. A power dependence of $\alpha = \alpha_0 \cdot 10^{P/20}$ is plotted alongside the data, for different values of α_0 (colours).

The results of the fit using the adiabatic model are also shown in Fig. 22, alongside power dependence curves $\alpha = \alpha_0 \cdot 10^{P/20}$, for different values of α_0 (indicated by the colours). This power dependence was used to describe replicas in conductance features, and matched the experimental data well up to large powers. However, this dependence does not accurately describe the trend of the values of α obtained from supercurrent measurements, for any value of α_0. We speculate that the different power dependence could originate from the finite size of the junction region, such that the electromagnetic field has local variations across the length of the junction. This situation would result in a time-varying bias dropping across different parts of the device, not just at the interface of the leads and junction as assumed by the Tien–Gordon model [1]. The complexity of the electromagnetic field distribution on the surface of the chip, and how it changes with power, might account for this power dependence. In addition, at large values of microwave power we observe deviations from the adiabatic model, namely dips in the supercurrent [see Fig. 5(d) in the Main Text]. These additional features indicate a non-thermal ABS occupation [12] (see Supplementary Note 13), which results in distortions to the CPR. Values of α obtained at large powers may therefore be unrepresentative, since they attempt to account for these additional distortions from within the adiabatic model.

We note that the exact power dependence of the fitted values for α does not change the result: the coupling strength to the supercurrent, obtained from measurements of the CPR, was much lower than the equivalent values from replicas in conductance features. This is inconsistent with conductance replicas originating from FASs in the junction.

SUPPLEMENTARY NOTE 13: NON-THERMAL ABS OCCUPATION

At large applied microwave power, the measured CPR deviated from the fitted curve using the adiabatic model. At some values of the perpendicular magnetic field $B_\perp$, corresponding to certain phase values φ, the measured switching current was closer to zero than expected from the adiabatic model. This is interpreted as a non-thermal occupation of ABSs in the SNS junction, due to excitations driven by the microwave field. A microwave photon can induce a transition when the excitation energy $2E_\text{A}$ is an integer multiple of the photon energy hf. Since E_A depends on the phase difference φ, absorption is expected only at specific φ for a given frequency f. This is schematically shown in Fig. 23(a), for the case of $\tau = 0.84$. The current carried by an excited ABS is equal and opposite to that in the ground state, resulting in a suppression in the average measured current. For large drive powers, multi-photon processes are possible, and transitions can occur into or out of ABSs from the quasiparticle continuum. Excitation is most likely to occur close to $\varphi = \pi$, since this is where $2E_\text{A}$ is minimised. This is particularly true for highly transmissive ABSs, where the separation of the ABS from the superconducting gap edge can be large. To describe the impact of these different microwave-induced transitions on the CPR, we employed the theory of Ref. [12, 15]. This theory, which is based on non-equilibrium Green's functions techniques, describes the CPR of a single channel superconducting point contact for arbitrary junction transparency (τ) and strength of the coupling between the microwave field and the Josephson current ($\alpha = \text{e}V_\text{MW}/hf$). Figure 23(b) shows the simulated CPR for microwave irradiation of $hf = 0.19\Delta$, corresponding to a frequency of 9.20 GHz, for increasing α up to 1. The full model (solid lines) deviates from the adiabatic theory (dashed lines) for $\alpha \gtrapprox 0.6$, consistent with the experimental observation.

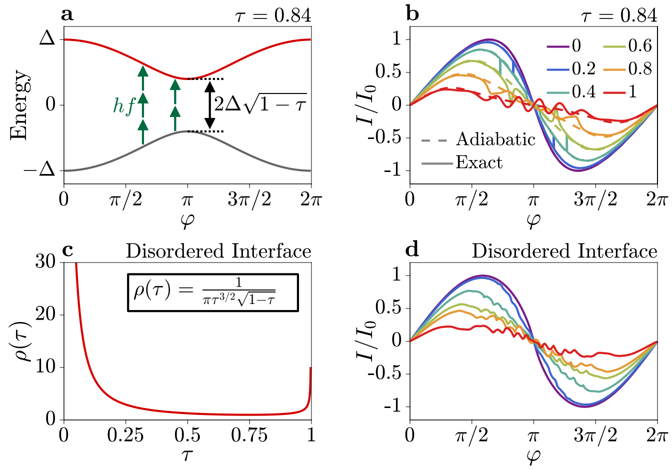

Supplementary Fig. 23. **Non-thermal occupation of Andreev bound states (ABSs).** **(a)** ABS spectrum for transmission $\tau = 0.84$. Transitions (green) from occupied (grey) to unoccupied (red) states can occur close to $\varphi = \pi$ by absorption of microwave photons with energy hf. **(b)** Normalised current–phase relation for transmission $\tau = 0.84$ under increasing microwave field amplitude α. Deviations of exact model (solid line) from adiabatic theory (dashed lines) occur at some values of φ due to non-thermal occupation. **(c)** Distribution of channel transmissions for a planar Josephson junction with a disordered interface. The transmission distribution follows the equation in inset [14]. **(d)** Normalised current–phase relation for a junction modelled with a disordered interface, under increasing microwave field amplitude α [colour defined in (b)].

The simulated CPR considers transitions in a single mode of transmission $\tau = 0.84$, equal to the effective transmission of the junction. However, this does not consider the many modes present in the junction. Figure 23(c) shows a distribution of transmissions in an SNS junction with a disordered interface, following the relation $\rho(\tau) = 1/\pi\tau^{3/2}\sqrt{1-\tau}$ [14]. The transmission distribution was chosen to give a CPR at equilibrium which matched the experimental result. The evolution of the CPR under microwave irradiation is shown in Fig. 23(d). The suppression in switching current is less pronounced than in the single mode case, but occurs across a wider range of φ. The experimental data shows strong suppression across a wide range of φ, suggesting that the SNS junction is between the two extremes outlined in Fig. 23. This is consistent with a junction containing many modes, some of which have a high transmission.

SUPPLEMENTARY REFERENCES

[1] Tien, P. K. & Gordon, J. P. Multiphoton process observed in the interaction of microwave fields with the tunneling between superconductor films. *Phys. Rev.* **129**, 647–651 (1963).

[2] Tinkham, M. *Introduction to Superconductivity* (Dover Publications, 2004), 2 edn.

[3] Platero, G. & Aguado, R. Photon-assisted transport in semiconductor nanostructures. *Phys. Rep.* **395**, 1–157 (2004).

[4] Peters, O. *et al.* Resonant Andreev reflections probed by photon-assisted tunnelling at the atomic scale. *Nat. Phys.* **16**, 1222–1226 (2020).

[5] Kot, P. *et al.* Microwave-assisted tunneling and interference effects in superconducting junctions under fast driving signals. *Phys. Rev. B* **101**, 134507 (2020).

[6] Park, S. *et al.* Steady Floquet–Andreev states in graphene Josephson junctions. *Nature* **603**, 421–426 (2022).

[7] Peltonen, J. T., Muhonen, J. T., Meschke, M., Kopnin, N. B. & Pekola, J. P. Magnetic-field-induced stabilization of nonequilibrium superconductivity in a normal-metal/insulator/superconductor junction. *Phys. Rev. B* **84**, 220502 (2011).

[8] Beenakker, C. W. J. & van Houten, H. Josephson current through a superconducting quantum point contact shorter than the coherence length. *Phys. Rev. Lett.* **66**, 3056–3059 (1991).

[9] Spanton, E. M. *et al.* Current–phase relations of few-mode InAs nanowire Josephson junctions. *Nat. Phys.* **13**, 1177–1181 (2017).

[10] Nichele, F. *et al.* Relating Andreev bound states and supercurrents in hybrid Josephson junctions. *Phys. Rev. Lett.* **124**, 226801 (2020).

[11] Barone, A. & Paternò, G. High Frequency Properties and Applications of the Josephson Effect. In *Physics and Applications of the Josephson Effect*, 291–353 (1982).

[12] Bergeret, F. S., Virtanen, P., Ozaeta, A., Heikkilä, T. T. & Cuevas, J. C. Supercurrent and Andreev bound state dynamics in superconducting quantum point contacts under microwave irradiation. *Phys. Rev. B* **84**, 054504 (2011).

[13] Dou, Z. *et al.* Microwave photoassisted dissipation and supercurrent of a phase-biased graphene-superconductor ring. *Phys. Rev. Res.* **3**, L032009 (2021).
[14] Schep, K. M. & Bauer, G. E. W. Transport through dirty interfaces. *Phys. Rev. B* **56**, 15860–15872 (1997).
[15] Virtanen, P., Heikkilä, T. T., Bergeret, F. S. & Cuevas, J. C. Theory of microwave-assisted supercurrent in diffusive SNS junctions. *Phys. Rev. Lett.* **104**, 247003 (2010).
